# Supplementary material for: Molecular Characterization of Portuguese Patients with Hereditary Cerebellar Ataxia
Source: Cells. 2022 Mar 12;11(6):981. doi: 10.3390/cells11060981 (PMC8946949; doi:10.3390/cells11060981)
Supplement: Supplementary file 1 [file cells-11-00981-s001.zip › Figure1_supporting info.pdf]

Supplementary figure S1

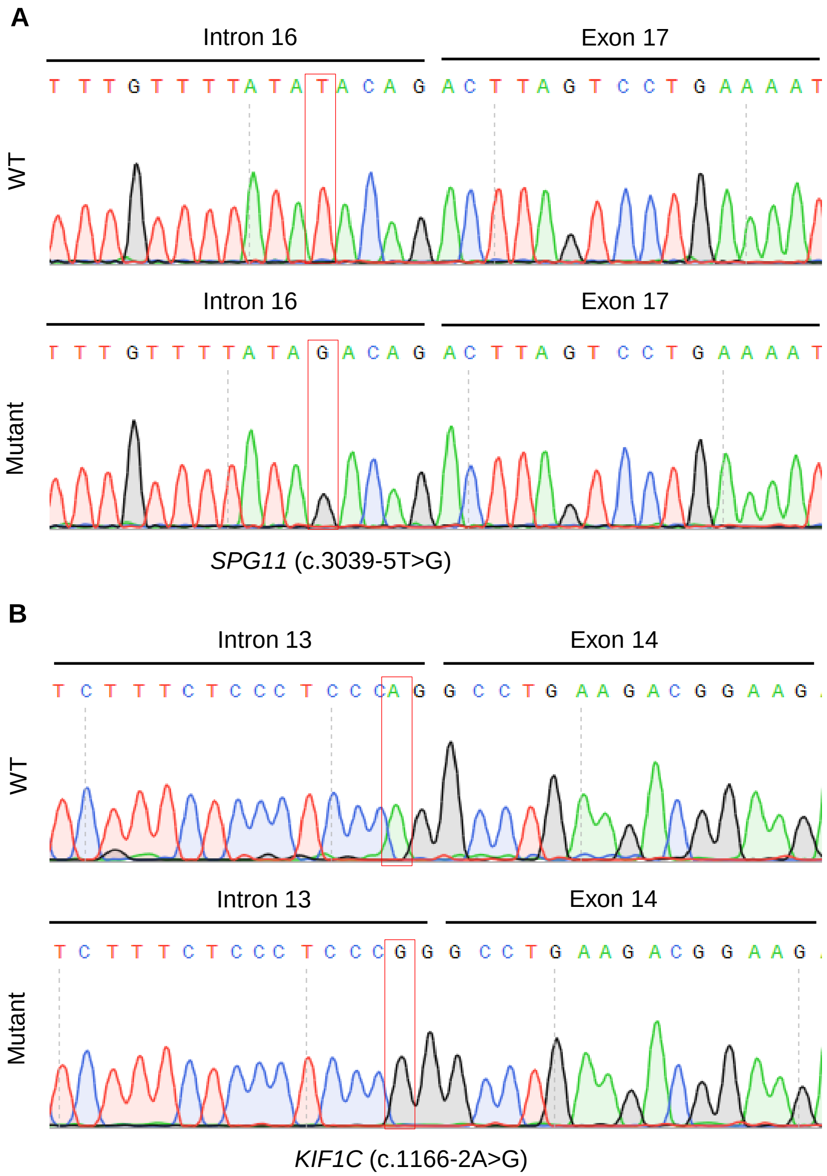

Supplementary figure S1. Electropherogram with the position of the *SPG11* (c.3039-5T>G) **(A)** and *KIF1C* (c.1166-2A>G) **(B)** splice-site variants boxed.
