# Supplementary material for: Molecular Characterization of Portuguese Patients with Hereditary Cerebellar Ataxia
Source: Cells. 2022 Mar 12;11(6):981. doi: 10.3390/cells11060981 (PMC8946949; doi:10.3390/cells11060981)
Supplement: Supplementary file 1 [file cells-11-00981-s001.zip › Table_S2_revised.pdf]

Table S2. Molecular functions and disease mechanisms associated with recessive cerebellar ataxia genes and encoded proteins, grouped by main clinical phenotype.

Gene	Protein	Subcellular localization	Molecular function(s)	Associated disease(s)	Disease mechanim(s)	References
**Spastic ataxia**						
SACS	Sacsin	Cytoplasm; Mitochondria	Co-chaperone of Hsp70 chaperone machinery; regulator of mitochondria dynamics; maintenance of cytoskeleton network	ARSACS	Loss-of-function: reduced chaperone activity, and altered mitochondria and neurofilament networks caused by sacsin depletion	[1-4]
KIF1C	Kinesin family member 1C (KIF1C)	Cytoskeleton/ microtubules	Anterograde movement of vesicles; retrograde transport from Golgi to ER	SPAX2; SPG58	Loss-of-function / gain-of-function? (functional studies are needed for confirmation)	[5-7]
ANO10	Anoctamin-10 (ANO10)	Plasma membrane	Regulates intracellular calcium signals	SCAR10/ ARCA3	Loss-of-function? (functional studies are needed for confirmation)	[8,9]
SPG11	Spatacsin	Cytoplasm; ER; Vesicles	Role in neuronal axonal growth, intracellular cargo trafficking and lysosome function	SPG11; ALS5; CMTD	Loss-of-function: impaired axonal transport and neurite outgrowth, and endolysosomal abnormalities due to spatacsin dysfunction	[10-14]
SYNE1	SYNE1/Nesprin-1	Nuclear envelope	Structural protein that links the nuclear envelope to the actin cytoskeleton (LINC complex)	ARCA1/ SCAR8; AMC; EDMD	Loss-of-function: nuclear morphology defects and defective synaptic transmission caused by nesprin-1 insufficiency	[15-18]
**Ataxia and neuropathy**						
POLG	DNA polymerase subunit gamma 1 (POLG)	Mitochondria	Involved in the replication of mtDNA and mtDNA repair	SANDO; SCAE; MDS; PEO	Loss-of-function: reduced POLG DNA polymerase activity and DNA-binding	[19-24]
**Ataxia with oculomotor apraxia (AOA)**						
SETX	Senataxin	Nucleus; Cytoplasm	Probable RNA/DNA helicase involved in transcription regulation, mRNA splicing and DNA damage response	AOA2; ALS4	Loss-of-function: altered gene expression and mRNA processing caused by senataxin disrupted function; Toxic gain-of-function: increased susceptibility to DNA-damaging agents	[25-28]

Gene	Protein	Subcellular localization	Molecular function(s)	Associated disease(s)	Disease mechanim(s)	References
PNKP	Polynucleotide kinase 3' phosphatase (PNKP)	Nucleus	Involved in DNA damage repair and cell cycle regulation	AOA4; MCSZ; CMTD	Loss-of-function: impaired DNA repair and cell cycle dynamics caused by hypomorphic PNKP	[29-32]
**Ataxia and dystonia**						
HEXB	Hexosaminidase subunit beta (HEXB)	Lysosome	Catalyzes the degradation of the GM2 ganglioside, and other molecules, in the lysosome	Sandhoff disease	Loss-of-function: accumulation of GM2 gangliosides and lysosomal abnormalities	[33,34]
**Ataxia with cognitive impairment**						
FA2H	Fatty acid 2-hydroxylase (FA2H)	ER membrane	Catalyzes the synthesis of 2-hydroxysphingolipids (myelin lipids)	SPG35	Loss-of-function: decreased hydroxylation of myelin lipid components	[35,36]

AMC3, arthrogryposis multiplex congenita; ALS, amyotrophic lateral sclerosis; AOA, ataxia and oculomotor apraxia; ARSACS, autosomal recessive spastic ataxia of Charlevoix-Saguenay; CMTD, Charcot-Marie-Tooth disease; EA, episodic ataxia; EDMD, Emery-Dreifuss muscular dystrophy; ER, endoplasmic reticulum; LINC, linker of nucleoskeleton and cytoskeleton; MCSZ, microcephaly, seizures, and developmental delay; MIRAS, mitochondrial recessive ataxia syndrome; MDS, mitochondrial DNA depletion syndrome; mtDNA, mitochondrial DNA; OA, oculomotor apraxia; SANDO, sensory ataxic neuropathy with dysarthria and ophthalmoparesis; SCAE, spinocerebellar ataxia with epilepsy; SPG, spastic paraplegia; PEO, progressive external ophthalmoplegia; WT, wild-type.

References

1. Girard, M.; Lariviere, R.; Parfitt, D.A.; Deane, E.C.; Gaudet, R.; Nossova, N.; Blondeau, F.; Prenosil, G.; Vermeulen, E.G.; Duchen, M.R.; et al. Mitochondrial dysfunction and Purkinje cell loss in autosomal recessive spastic ataxia of Charlevoix-Saguenay (ARSACS). *Proc Natl Acad Sci U S A* **2012**, *109*, 1661-1666, doi:10.1073/pnas.1113166109.
2. Lariviere, R.; Gaudet, R.; Gentil, B.J.; Girard, M.; Conte, T.C.; Minotti, S.; Leclerc-Desaulniers, K.; Gehring, K.; McKinney, R.A.; Shoubridge, E.A.; et al. Sacs knockout mice present pathophysiological defects underlying autosomal recessive spastic ataxia of Charlevoix-Saguenay. *Hum Mol Genet* **2015**, *24*, 727-739, doi:10.1093/hmg/ddu491.
3. Parfitt, D.A.; Michael, G.J.; Vermeulen, E.G.; Prodromou, N.V.; Webb, T.R.; Gallo, J.M.; Cheetham, M.E.; Nicoll, W.S.; Blatch, G.L.; Chapple, J.P. The ataxia protein sacsin is a functional co-chaperone that protects against polyglutamine-expanded ataxin-1. *Hum Mol Genet* **2009**, *18*, 1556-1565, doi:10.1093/hmg/ddp067.
4. Engert, J.C.; Berube, P.; Mercier, J.; Dore, C.; Lepage, P.; Ge, B.; Bouchard, J.P.; Mathieu, J.; Melancon, S.B.; Schalling, M.; et al. ARSACS, a spastic ataxia common in northeastern Quebec, is caused by mutations in a new gene encoding an 11.5-kb ORF. *Nat Genet* **2000**, *24*, 120-125, doi:10.1038/72769.
5. Gabrych, D.R.; Lau, V.Z.; Niwa, S.; Silverman, M.A. Going Too Far Is the Same as Falling Short(dagger): Kinesin-3 Family Members in Hereditary Spastic Paraplegia. *Front Cell Neurosci* **2019**, *13*, 419, doi:10.3389/fncel.2019.00419.

6. Dor, T.; Cinnamon, Y.; Raymond, L.; Shaag, A.; Bouslam, N.; Bouhouche, A.; Gaussen, M.; Meyer, V.; Durr, A.; Brice, A.; et al. KIF1C mutations in two families with hereditary spastic paraparesis and cerebellar dysfunction. *J Med Genet* **2014**, *51*, 137-142, doi:10.1136/jmedgenet-2013-102012.
7. Alfares, A.; Alfadhel, M.; Wani, T.; Alsahli, S.; Alluhaydan, I.; Al Mutairi, F.; Alothaim, A.; Albalwi, M.; Al Subaie, L.; Alturki, S.; et al. A multicenter clinical exome study in unselected cohorts from a consanguineous population of Saudi Arabia demonstrated a high diagnostic yield. *Mol Genet Metab* **2017**, *121*, 91-95, doi:10.1016/j.ymgme.2017.04.002.
8. Balreira, A.; Boczonadi, V.; Barca, E.; Pyle, A.; Bansagi, B.; Appleton, M.; Graham, C.; Hargreaves, I.P.; Rasic, V.M.; Lochmuller, H.; et al. ANO10 mutations cause ataxia and coenzyme Q(1)(0) deficiency. *J Neurol* **2014**, *261*, 2192-2198, doi:10.1007/s00415-014-7476-7.
9. Vermeer, S.; Hoischen, A.; Meijer, R.P.; Gilissen, C.; Neveling, K.; Wieskamp, N.; de Brouwer, A.; Koenig, M.; Anheim, M.; Assoum, M.; et al. Targeted next-generation sequencing of a 12.5 Mb homozygous region reveals ANO10 mutations in patients with autosomal-recessive cerebellar ataxia. *Am J Hum Genet* **2010**, *87*, 813-819, doi:10.1016/j.ajhg.2010.10.015.
10. Perez-Branguli, F.; Mishra, H.K.; Prots, I.; Havlicek, S.; Kohl, Z.; Saul, D.; Rummel, C.; Dorca-Arevalo, J.; Regensburger, M.; Graef, D.; et al. Dysfunction of spatacsin leads to axonal pathology in SPG11-linked hereditary spastic paraplegia. *Hum Mol Genet* **2014**, *23*, 4859-4874, doi:10.1093/hmg/ddu200.
11. Renvoise, B.; Chang, J.; Singh, R.; Yonekawa, S.; FitzGibbon, E.J.; Mankodi, A.; Vanderver, A.; Schindler, A.; Toro, C.; Gahl, W.A.; et al. Lysosomal abnormalities in hereditary spastic paraplegia types SPG15 and SPG11. *Ann Clin Transl Neurol* **2014**, *1*, 379-389, doi:10.1002/acn3.64.
12. Stevanin, G.; Azzedine, H.; Denora, P.; Boukhris, A.; Tazir, M.; Lossos, A.; Rosa, A.L.; Lerer, I.; Hamri, A.; Alegria, P.; et al. Mutations in SPG11 are frequent in autosomal recessive spastic paraplegia with thin corpus callosum, cognitive decline and lower motor neuron degeneration. *Brain* **2008**, *131*, 772-784, doi:10.1093/brain/awm293.
13. Orlacchio, A.; Babalini, C.; Borreca, A.; Patrono, C.; Massa, R.; Basaran, S.; Munhoz, R.P.; Rogaeva, E.A.; St George-Hyslop, P.H.; Bernardi, G.; et al. SPATACSIN mutations cause autosomal recessive juvenile amyotrophic lateral sclerosis. *Brain* **2010**, *133*, 591-598, doi:10.1093/brain/awp325.
14. Montecchiani, C.; Pedace, L.; Lo Giudice, T.; Casella, A.; Mearini, M.; Gaudiello, F.; Pedroso, J.L.; Terracciano, C.; Caltagirone, C.; Massa, R.; et al. ALS5/SPG11/KIAA1840 mutations cause autosomal recessive axonal Charcot-Marie-Tooth disease. *Brain* **2016**, *139*, 73-85, doi:10.1093/brain/awv320.
15. Synofzik, M.; Smets, K.; Mallaret, M.; Di Bella, D.; Gallenmuller, C.; Baets, J.; Schulze, M.; Magri, S.; Sarto, E.; Mustafa, M.; et al. SYNE1 ataxia is a common recessive ataxia with major non-cerebellar features: a large multi-centre study. *Brain* **2016**, *139*, 1378-1393, doi:10.1093/brain/aww079.
16. Zhang, Q.; Bethmann, C.; Worth, N.F.; Davies, J.D.; Wasner, C.; Feuer, A.; Ragnauth, C.D.; Yi, Q.; Mellad, J.A.; Warren, D.T.; et al. Nesprin-1 and -2 are involved in the pathogenesis of Emery Dreifuss muscular dystrophy and are critical for nuclear envelope integrity. *Hum Mol Genet* **2007**, *16*, 2816-2833, doi:10.1093/hmg/ddm238.
17. Gros-Louis, F.; Dupre, N.; Dion, P.; Fox, M.A.; Laurent, S.; Verreault, S.; Sanes, J.R.; Bouchard, J.P.; Rouleau, G.A. Mutations in SYNE1 lead to a newly discovered form of autosomal recessive cerebellar ataxia. *Nat Genet* **2007**, *39*, 80-85, doi:10.1038/ng1927.
18. Razafsky, D.; Hodzic, D. A variant of Nesprin1 giant devoid of KASH domain underlies the molecular etiology of autosomal recessive cerebellar ataxia type I. *Neurobiol Dis* **2015**, *78*, 57-67, doi:10.1016/j.nbd.2015.03.027.

19. Luoma, P.T.; Luo, N.; Loscher, W.N.; Farr, C.L.; Horvath, R.; Wanschitz, J.; Kiechl, S.; Kaguni, L.S.; Suomalainen, A. Functional defects due to spacer-region mutations of human mitochondrial DNA polymerase in a family with an ataxia-myopathy syndrome. *Hum Mol Genet* **2005**, *14*, 1907-1920, doi:10.1093/hmg/ddi196.
20. Schaller, A.; Hahn, D.; Jackson, C.B.; Kern, I.; Chardot, C.; Belli, D.C.; Gallati, S.; Nuoffer, J.M. Molecular and biochemical characterisation of a novel mutation in POLG associated with Alpers syndrome. *BMC Neurol* **2011**, *11*, 4, doi:10.1186/1471-2377-11-4.
21. Van Goethem, G.; Dermaut, B.; Lofgren, A.; Martin, J.J.; Van Broeckhoven, C. Mutation of POLG is associated with progressive external ophthalmoplegia characterized by mtDNA deletions. *Nat Genet* **2001**, *28*, 211-212, doi:10.1038/90034.
22. Naviaux, R.K.; Nguyen, K.V. POLG mutations associated with Alpers' syndrome and mitochondrial DNA depletion. *Ann Neurol* **2004**, *55*, 706-712, doi:10.1002/ana.20079.
23. Winterthun, S.; Ferrari, G.; He, L.; Taylor, R.W.; Zeviani, M.; Turnbull, D.M.; Engelsen, B.A.; Moen, G.; Bindoff, L.A. Autosomal recessive mitochondrial ataxic syndrome due to mitochondrial polymerase gamma mutations. *Neurology* **2005**, *64*, 1204-1208, doi:10.1212/01.WNL.0000156516.77696.5A.
24. Vissing, J.; Ravn, K.; Danielsen, E.R.; Duno, M.; Wibrand, F.; Wevers, R.A.; Schwartz, M. Multiple mtDNA deletions with features of MNGIE. *Neurology* **2002**, *59*, 926-929, doi:10.1212/wnl.59.6.926.
25. Airoldi, G.; Guidarelli, A.; Cantoni, O.; Panzeri, C.; Vantaggiato, C.; Bonato, S.; Grazia D'Angelo, M.; Falcone, S.; De Palma, C.; Tonelli, A.; et al. Characterization of two novel SETX mutations in AOA2 patients reveals aspects of the pathophysiological role of senataxin. *Neurogenetics* **2010**, *11*, 91-100, doi:10.1007/s10048-009-0206-0.
26. Fogel, B.L.; Cho, E.; Wahnich, A.; Gao, F.; Becherel, O.J.; Wang, X.; Fike, F.; Chen, L.; Criscuolo, C.; De Michele, G.; et al. Mutation of senataxin alters disease-specific transcriptional networks in patients with ataxia with oculomotor apraxia type 2. *Hum Mol Genet* **2014**, *23*, 4758-4769, doi:10.1093/hmg/ddu190.
27. Moreira, M.C.; Klur, S.; Watanabe, M.; Nemeth, A.H.; Le Ber, I.; Moniz, J.C.; Tranchant, C.; Aubourg, P.; Tazir, M.; Schols, L.; et al. Senataxin, the ortholog of a yeast RNA helicase, is mutant in ataxia-ocular apraxia 2. *Nat Genet* **2004**, *36*, 225-227, doi:10.1038/ng1303.
28. Chen, Y.Z.; Bennett, C.L.; Huynh, H.M.; Blair, I.P.; Puls, I.; Irobi, J.; Dierick, I.; Abel, A.; Kennerson, M.L.; Rabin, B.A.; et al. DNA/RNA helicase gene mutations in a form of juvenile amyotrophic lateral sclerosis (ALS4). *Am J Hum Genet* **2004**, *74*, 1128-1135, doi:10.1086/421054.
29. Bras, J.; Alonso, I.; Barbot, C.; Costa, M.M.; Darwent, L.; Orme, T.; Sequeiros, J.; Hardy, J.; Coutinho, P.; Guerreiro, R. Mutations in PNKP cause recessive ataxia with oculomotor apraxia type 4. *Am J Hum Genet* **2015**, *96*, 474-479, doi:10.1016/j.ajhg.2015.01.005.
30. Pedroso, J.L.; Rocha, C.R.; Macedo-Souza, L.I.; De Mario, V.; Marques, W., Jr.; Barsottini, O.G.; Bulle Oliveira, A.S.; Menck, C.F.; Kok, F. Mutation in PNKP presenting initially as axonal Charcot-Marie-Tooth disease. *Neurol Genet* **2015**, *1*, e30, doi:10.1212/NXG.0000000000000030.
31. Reynolds, J.J.; Walker, A.K.; Gilmore, E.C.; Walsh, C.A.; Caldecott, K.W. Impact of PNKP mutations associated with microcephaly, seizures and developmental delay on enzyme activity and DNA strand break repair. *Nucleic Acids Res* **2012**, *40*, 6608-6619, doi:10.1093/nar/gks318.
32. Shen, J.; Gilmore, E.C.; Marshall, C.A.; Haddadin, M.; Reynolds, J.J.; Eyaid, W.; Bodell, A.; Barry, B.; Gleason, D.; Allen, K.; et al. Mutations in PNKP cause microcephaly, seizures and defects in DNA repair. *Nat Genet* **2010**, *42*, 245-249, doi:10.1038/ng.526.

33. Bolhuis, P.A.; Oonk, J.G.; Kamp, P.E.; Ris, A.J.; Michalski, J.C.; Overdijk, B.; Reuser, A.J. Ganglioside storage, hexosaminidase lability, and urinary oligosaccharides in adult Sandhoff's disease. *Neurology* **1987**, *37*, 75-81, doi:10.1212/wnl.37.1.75.
34. Redonnet-Vernhet, I.; Mahuran, D.J.; Salvayre, R.; Dubas, F.; Levade, T. Significance of two point mutations present in each HEXB allele of patients with adult GM2 gangliosidosis (Sandhoff disease) homozygosity for the Ile207-->Val substitution is not associated with a clinical or biochemical phenotype. *Biochim Biophys Acta* **1996**, *1317*, 127-133, doi:10.1016/s0925-4439(96)00044-0.
35. Dick, K.J.; Eckhardt, M.; Paisan-Ruiz, C.; Alshehhi, A.A.; Proukakis, C.; Sibtain, N.A.; Maier, H.; Sharifi, R.; Patton, M.A.; Bashir, W.; et al. Mutation of FA2H underlies a complicated form of hereditary spastic paraplegia (SPG35). *Hum Mutat* **2010**, *31*, E1251-1260, doi:10.1002/humu.21205.
36. Edvardson, S.; Hama, H.; Shaag, A.; Gomori, J.M.; Berger, I.; Soffer, D.; Korman, S.H.; Taustein, I.; Saada, A.; Elpeleg, O. Mutations in the fatty acid 2-hydroxylase gene are associated with leukodystrophy with spastic paraparesis and dystonia. *Am J Hum Genet* **2008**, *83*, 643-648, doi:10.1016/j.ajhg.2008.10.010.
