# Supplementary material for: Molecular Characterization of Portuguese Patients with Hereditary Cerebellar Ataxia
Source: Cells. 2022 Mar 12;11(6):981. doi: 10.3390/cells11060981 (PMC8946949; doi:10.3390/cells11060981)
Supplement: Supplementary file 1 [file cells-11-00981-s001.zip › Table_S4.pdf]

Table S4. Protein domain location of variants identified.

Gene	cDNA	Protein	Protein domain
SACS	c.8148del	p.Pro2717Glnfs*22	Sacsin-repeating region with homology with Hsp90
	c.1373C>T	p.Thr458Ile	Sacsin-repeating region with homology with Hsp90
	c. 5440_5449del	Glu1814Argfs*14	Sacsin-repeating region with homology with Hsp90
	c.3066del	Asn1025Metfs*10	Non-specific domain
	c.2656_2666del	Gln886*	Non-specific domain
	c.814C>T	p.Arg272Cys	Sacsin-repeating region with homology with Hsp90
KIF1C	c.393_396del	Ser131Argfs*13	Motor domain
	c.1166-2A>G	p.Gly389Aspfs*29	Coiled-coil domain
AN010	c.132dup	p.Asp45Argfs*9	Non-specific domain
SPG11	c.1951C>T	Arg651*	Non-specific domain
	c.3039-5T>G	Leu1014Glnfs*3	Non-specific domain
SYNE1	c.17270C>G	p.Thr5757Arg	Spectrin repeat domain
CACNA1A	c.4996C>G	p.Arg1666Gly	Transmembrane domain
KIF1A	c.761G>A	p.Arg254Gln	Kinesin motor domain
POLG	c.2243G>C	p.Trp748Ser	RNA-directed RNA polymerase, C-terminal domain
SETX	c.6139G>T	p.Gly2047Cys	DNA2/NAM7 helicase domain
PNKP	c.1123G>T	Gly375Trp	Kinase domain
	c.1253_1269dup	Thr424Glyfs*49	Kinase domain
	c.1123G>T	p.Gly375Trp	Kinase domain
	c.1221_1223del	p.Thr408del	Kinase domain
	c.1123G>T	p.Gly375Trp	Kinase domain
HEXB	c.1514G>A	p.Arg505Gln	Glycoside Hydrolase Catalytic Domain
ATP1A3	c.374T>A	p.Val125Glu	Transmembrane domain
FA2H	c.619_620del	p.Thr207Glyfs*105	Non-specific domain
