# Supplementary material for: Molecular Characterization of Portuguese Patients with Hereditary Cerebellar Ataxia
Source: Cells. 2022 Mar 12;11(6):981. doi: 10.3390/cells11060981 (PMC8946949; doi:10.3390/cells11060981)
Supplement: Supplementary file 1 [file cells-11-00981-s001.zip › Table_S5.pdf]

Table S5. RNA and protein expression data obtained from the Human Protein Atlas for genes and respective proteins identified in this study.

	RNA levels				Protein levels	
**Genes**	**Brain enriched**	**Cortex**	**Cerebellum**	**Spinal cord**	**Cortical neuronal cells**	**Cerebellar Purkinje cells**
KIF1A	Yes	109.0	18.5	20.2	-	-
POLG	No	6.0	4.5	5.8	Low	High
SETX	No	6.8	9.8	8.1	Medium	Medium
PNKP	No	16.4	11.0	10.5	High	Medium
AN010	No	21.0	23.8	20.0	High	Medium
CACNA1A	Yes	25.7	91.8	6.7	High	ND
SACS	No	17.2	8.8	4.1	Medium	Medium
SPG11	No	21.4	23.3	18.8	Medium	Low
KIF1C	Yes	211.6	203.0	225.5	Low	Medium
SYNE1	Yes	72.3	150.2	40.6	High	High
HEXB	No	32.8	23.3	25.3	ND	ND
ATP1A3	Yes	634.7	84.4	10	ND	ND
FA2H	Yes	61.7	67.1	75.5	ND	ND

Data was obtained from the The Human Protein Atlas [1]. The RNA values are expressed as the normalized transcripts per Kb million (nTPM) expression levels from the consensus dataset (combines the HPA and GTEx transcriptomics datasets using the internal normalization pipeline). Protein levels are qualitative, achieved by evaluation of antibody staining, RNA-seq data, and annotated protein expression data. ND, not detected.

Reference

1. Uhlen, M.; Fagerberg, L.; Hallstrom, B.M.; Lindskog, C.; Oksvold, P.; Mardinoglu, A.; Sivertsson, A.; Kampf, C.; Sjostedt, E.; Asplund, A.; et al. Proteomics. Tissue-based map of the human proteome. *Science* **2015**, *347*, 1260419, doi:10.1126/science.1260419.
